# Development of a Convolutional Neural Network Based Skull Segmentation in MRI Using Standard Tesselation Language Models

**DOI:** 10.3390/jpm11040310

**Published:** 2021-04-16

**Authors:** Rodrigo Dalvit Carvalho da Silva, Thomas Richard Jenkyn, Victor Alexander Carranza

**Affiliations:** 1 Craniofacial Injury and Concussion Research Laboratory, Western University, London, ON N6A 3K7, Canada; tjenkyn@uwo.ca (T.R.J.); vcarranz@uwo.ca (V.A.C.); 2 Faculty of Engineering, School of Biomedical Engineering, Western University, London, ON N6A 3K7, Canada; 3 Department of Mechanical and Materials Engineering, Western University, London, ON N6A 3K7, Canada; 4 Faculty of Health Sciences, School of Kinesiology, Western University, London, ON N6A 3K7, Canada; 5 Wolf Orthopaedic Biomechanics Laboratory, Fowler Kennedy Sport Medicine Clinic, London, ON N6A 3K7, Canada; 6 Faculty of Engineering, School of Biomedical Engineering, Collaborative Specialization in Musculoskeletal Health Research, and Bone and Joint Institute, Western University, London, ON N6A 3K7, Canada

**Keywords:** Convolutional Neural Network, Standard Tessellation Language, Image Segmentation, MRI, CT

## Abstract

Segmentation is crucial in medical imaging analysis to help extract regions of interest (ROI) from different imaging modalities. The aim of this study is to develop and train a 3D convolutional neural network (CNN) for skull segmentation in magnetic resonance imaging (MRI). 58 gold standard volumetric labels were created from computed tomography (CT) scans in standard tessellation language (STL) models. These STL models were converted into matrices and overlapped on the 58 corresponding MR images to create the MRI gold standards labels. The CNN was trained with these 58 MR images and a mean ± standard deviation (SD) Dice similarity coefficient (DSC) of 0.7300 ± 0.04 was achieved. A further investigation was carried out where the brain region was removed from the image with the help of a 3D CNN and manual corrections by using only MR images. This new dataset, without the brain, was presented to the previous CNN which reached a new mean ± SD DSC of 0.7826 ± 0.03. This paper aims to provide a framework for segmenting the skull using CNN and STL models, as the 3D CNN was able to segment the skull with a certain precision.

## 1. Introduction

Image segmentation is the process of partitioning an image into multiple sections to simplify the image into something more meaningful so that we can easily locate regions of interest (ROI). In medical imaging and analysis, these ROI, identified by the segmentation process in an image scanning system, can represent various structures in the body such as pathologies, tissues, bone, organs, prosthesis, and so forth.

Magnetic resonance imaging (MRI) and computed tomography (CT) are the most common medical image scanning system used to reveal relevant structures for automated processing of scanned data. Both techniques are excellent in providing non-invasive diagnostic images of organs and structures inside the body. However, CT is not favorable for routine anatomical imaging of the head since it exposes the patient to small doses of ionizing radiation each visit putting the patient at risk for developing diseases such as cancer. For instance, a study in [1] pointed out that the risk to develop leukemia and brain tumors increases with the radiation exposure from CT scans. On the contrary, MRI scans have difficulty identifying different tissues because of the low signal-to-noise ratio of MRI. Additionally, due to bones’ weak magnetic resonance signal, MRI scans struggle with differentiating bone tissue from other structures. Specifically, since different bone tissues have the tendency to differ more in appearance from one another than from the adjacent muscle tissue, segmentation approaches must be robust to account for the variations in the structure [2]. Thus, bone segmentation from MRI presents a challenging problem. Current biomedical imaging segmentation methods take advantage of deep learning with convolutional neural networks (CNNs) [3], as seen in [4] where they trained a large deep CNN to classify over 1 million high-resolution images with a top-1 and top-5 test set error rates of 37.5% and 17.0%, much better than the previous state-of-the-art technique.

In CNN, each layer contains various neurons fixed in subsequent layers and sharing weighted connections. During training, these layers extract features (such as horizontal or vertical edges) from the training images that allows CNN to perform certain tasks such as segmentation by recognizing these features in subsequent images. The advantage of CNN over other techniques is that convolutional image filters are learned and adapted in an automated process for a high-level description in the finest optimization process.

With recent advances in graphical processing units and improvements in computational efficiency, CNNs have achieved excellent results in biomedical image segmentation where deep learning approaches can be performed in an efficient and intelligent way. CNN has been extensively applied in musculoskeletal image segmentation tasks such as brain and spine segmentation [5], acute brain hemorrhage [6], vessel segmentation [7], skull stripping in brain MRI [8], knee bone and cartilage segmentation [9], segmentation of craniomaxillofacial bony structures [10], proximal femur segmentation [11], and cardiac image segmentation [12].

A review on deep learning techniques has been performed by Garcia et al [13] where the authors highlight a promising deep learning framework for segmentation tasks known as UNet [14]. UNet is a CNN which uses an encoding down-sampling path and an up-sampling decoding path for segmentation tasks to increase the resolution of the output, which has shown high performance when applied on biomedical images [7].

Although numerous MRI segmentation techniques are described in the literature, few have focused on segmenting the skull in MRI. One approach to segment the skull in MRI is mathematical morphology [15]. The authors describe a method where they first remove the brain by using a surface extractor algorithms and mask the scalp using thresholding and mathematical morphology. During the skull segmentation process, the authors use mathematical morphology to omit background voxels with similar intensities as the skull. Using thresholding and morphological operations, the inner and outer skull boundaries are identified, and the results are masked with the scalp and brain volumes to establish a closed and nonintersecting skull boundary. Applying this segmentation method to 44 images, the authors were able to achieve a mean dice coefficients of 0.7346, 0.6918, and 0.6337 for shifts CT of 1 mm, 2 mm, and 3 mm respectively.

Wang et al [16] utilized statistical shape information in 15 subjects, where the anatomy of interest is differentiated in the CT data by means of constructing an active shape model of the skull surfaces. The automatic landmarking on the coupled surfaces is optimized in statistical shape information by minimizing the description length that included the local thickness information. This method showed a dice coefficient of 0.7500 for one calvarium segmented. Support vector machine (SVM) combining local and global features are used in [17]. Feature vectors are constructed from each voxel in the image that is used as the first entry. The second input for this method uses a combination of intensities of a set of nearby voxels and statistical moments of the local surroundings. This feature vector is then introduced to a trained SVM that classifies the image as either a skull, or something else. By using SVM, the authors found a dice function mean of 0.7500 (0.68 minimum and 0.81 maximum) from 10 patients in a dataset of 40 patients.

Ref. [18] introduced a convolutional restricted Boltzmann machines (cRBM) for skull segmentation. This technique incorporates cRBM shape model into Statistical Parametric Mapping 8 (SPM8) segmentation framework [19,20] applied in 23 images. This method reached a median dice score for T1-weighted of 0.7344 and for T1-w + T2-w, 0.7446.

Most recently, [21] analyzed three methods of skull segmentation and identified multiple factors contributing to the enhancement of the standard of segmentation. Using a data set obtained from 10 patients, they concluded that improved skull segmentation was accomplished by FSL [22] and SPM12 [23], achieving a mean dice coefficient of 0.76 and 0.80 respectively.

These techniques present an effective method with a mean DSC of 0.75 for small datasets, however, for larger datasets or when extended to images collected from different MRI devices where the image suffers from noise and variation in the choice of parameter values, this effectiveness may be compromised.

Arguably, one of the most important components in machine learning and deep learning are the ground truth labels. Careful collection of data and high-quality ground truth labels that will be used to train and test a model is imperative for a successful deep learning project, but comes with a cost in computation energy and may become very time consuming [24]. Minnema et al [25] displayed a high overlap with gold standard segmentation by introducing a CNN for skull segmentation in CT scans. In the image processing step, a 3D surface model, which represents the label, was created in the standard tessellation language (STL) file format, a well-established method to represent 3D models [26,27,28,29]. To convert the files from CT to STL, segmentation of a high-quality gold standard STL model was performed manually by an experienced medical engineer. The results show a slight one voxel difference between the gold standard segmentation and the CNN segmentation, with a mean dice similarity coefficient of 0.9200.

**Figure 1:**
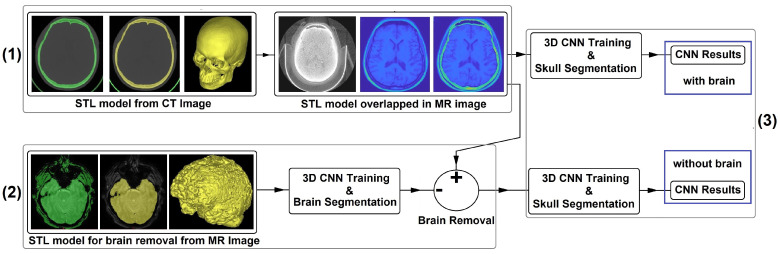
(**1**) STL models are produced from 58 CT scans and then overlapped with the MR images to create the first dataset. (**2**) 62 MR images are used to create brain STL models, and a brain segmentation algorithm is created. The brain segmentation algorithm is combined with manual corrections to remove the brain from dataset 1 to create dataset 2. (**3**) Finally, these 2 datasets are compared using the same CNN topology.

Therefore, this work aims to introduce the deep learning approach, more precisely UNet, for skull segmentation purpose where the ground truth labels are created from CT imaging using the STL representation file format. Figure 1 presents the schematic overview of the proposed study. First STL models are created from 58 CT scans. After being converted into matrices, these images are then overlapped with the MR images to create the gold standard and the first dataset. Then, using dataset 1, the first CNN is created. To improve the accuracy, 62 MR images are used to generate brain STL models. The models are then converted into matrices to create a set of brain gold standard and a second dataset. A brain segmentation algorithm using a second CNN is created and, through this CNN model and manual corrections, the brain is removed from dataset 1. Finally, this new dataset is presented again to the first CNN topology.

## 2. Materials and Methods

### 2.1. Dataset

We used the cancer imaging archive data collections (TCIA) [30] to search for reliable datasets that contain CT and MRI from the same patient and a minimum variation in the coronal, sagittal, and transverse plane. 58 volumetric CT and MR images were selected from four datasets to meet these criteria:

- CPTAC-GBM [31]—this dataset contains collection from the National Cancer Institute’s Clinical Proteomic Tumor Analysis Consortium Glioblastoma Multiform cohort. It contains CR, CT, MR, SC imaging modalities from 66 participants, totaling 164 studies;
- HNSCC [32,33,34]—this collection contains CT, MR, PT, RT, RTDOSE, RTPLAN, RTSTRUCT, from 627 subjects in a total of 1177 studies;
- TCGA-HNSC [35]—the cancer genome atlas head-neck squamous cell carcinoma data collection 479 studies from 227 participants from CT, MR, PET, RTDOSE, RTPLAN, RTSTRUCT modalities;
- ACRIN-FMISO [36,37,38]—the ACRIN 6684 multi-center clinical trial contains 423 studies applied in 45 participants using CT, MR, and PET modalities.

### 2.2. Data Processing I

**Figure 2:**
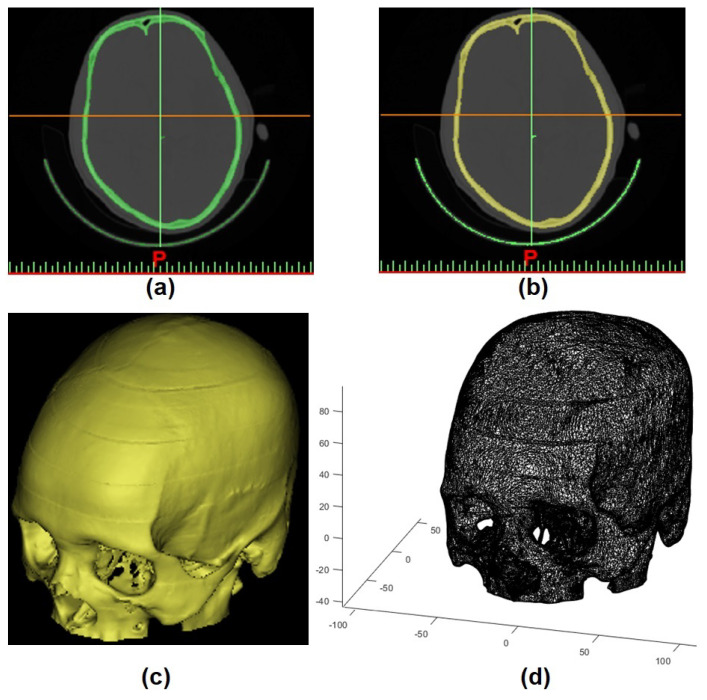
(**a**) thresholding applied in CT scan, (**b**) region growing, (**c**) 3D mesh model (STL model), and (**d**) STL model converted into matrix.

As this study aims to use CT scans to create ground truth labels, the first step was to generate the STL models. To perform this task, CT images were imported into Mimics Medical Imaging Software (Materialise, Leuven, Belgium). By using individual global thresholding in combination with manual corrections, the 3D model mesh was built, which allowed the STL model to be constructed (Figure 2a–c).

Then, to convert the geometric information (STL model) into a continuous domain (matrix), voxelisation was performed using [39] in MATLAB R2019B software (Figure 2d).

**Figure 3:**
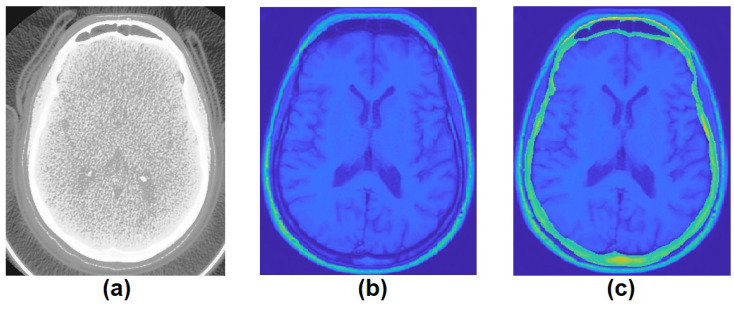
(**a**) CT scan, (**b**) MRI, and (**c**) STL model extracted from CT scan overlapped in MRI.

To generate the MRI labels, the STL models extracted from CT ground truth was overlapped into each MRI slice in 3-modal MRI (T1, T2, and FLAIR) using a combination of manual translations, rotations, and scaling. These manual alignments were followed by visual inspection and fine adjustment to ensure good quality (Figure 3). T2-weighted scans were included because the border between the skull and cerebro-spinal fluid (CSF) can be better delineated, as CSF appears bright in T2-weighted scans and has presented good results in [18,21].

Finally, all images were normalized between the range of 0 and 1 and then resized to 256 × 256 using nearest-neighbor interpolation method to improve the processing time.

### 2.3. Data Processing II

**Figure 4:**
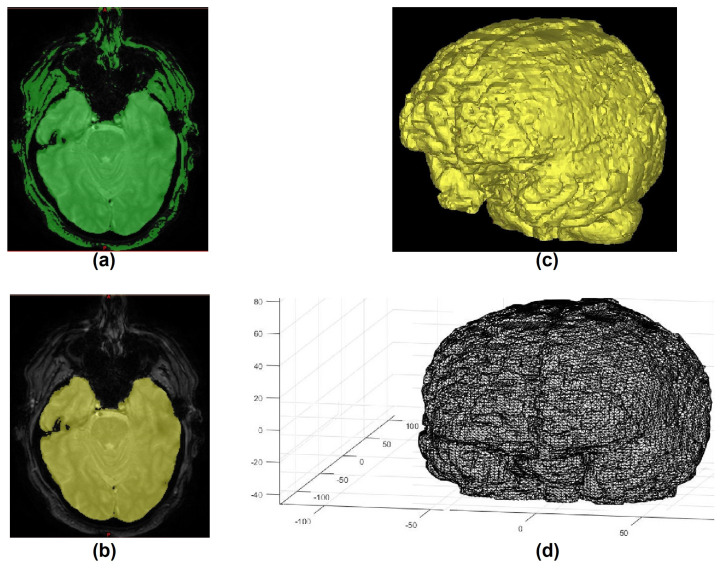
(**a**) thresholding applied in MRI, (**b**) region growing, (**c**) 3D mesh model (STL model), and (**d**) STL model converted into matrix.

To generate a second comparison, which can lead to an improvement in the accuracy, a reduction in the dataset information was performed by removing region of non-interest. The idea is to reduce the information content in the dataset by removing the gray and white matter. To perform this task, 62 volumetric MR images were randomly chosen and, in a similar manner explained in Section 2.2, brain gold standard labels were created from the MR image. The creation of brain labels is easily performed in MRI since the brain is easy to identify in magnetic resonance. The processing initially starts with the application of the thresholding, followed by region growing, and then creation and extraction of the STL models from the MRI (Figure 4).

### 2.4. CNN Architecture and Implementation Details

The deep learning framework chosen in this paper was UNet which was introduced by Ronneberger et al. [14]. This type of CNN was chosen because it works well with very few training images, yields more precise segmentation, and has been used in a number of recent biomedical image segmentation applications [5,9,10,11,40]. This network allows for a large number of feature channels in the upsampling procedure, which contribute in the propagation of context information to the highest resolution layers. The result is a more symmetric expansive path and a U-shaped architecture.

In our implementation, we adopted a 3D UNet initially developed for brain tumor segmentation in MRI [41]. To avoid class imbalance when using conventional cross entropy loss, a weighted multiclass dice loss function was used as the general loss of the network [42]. Table 1 shows the implementation parameter chosen for the skull segmentation. The parameters were chosen to avoid computational error, inprove the robustness and generalization ability of the CNN, and obtain a good accuracy for the training set explored in this work.

The CNN model was performed on Intel i7-9700 (3.00 GHz) workstations with 64 GB of ram, and two 8GB VRAM graphic cards from NVIDIA (RTX 2070 SUPER and RTX 2080). The code was implemented in MATLAB R2019B.

### 2.5. Model Performance Evaluation and Statistical Analysis

To evaluate the CNN segmentation, Dice Similarity Coefficient (DSC) [43], Symmetric Volume Difference (SVD) [44], Jaccard Similarity Coefficient (JSC) [45], Volumetric Overlap Error (VOE) [46], and Hausdorff distances (HD) [47] methods were used.

Dice similarity coefficient is a spatial overlap index that varies from the ranges 0, indicating no spatial overlap between two sets of binary segmentation results, to 1, indicating complete overlap [48]. SVD gives the symmetric difference of the shape and segmentation in terms of dice-based error metric. JSC is a similarity ratio which describes the intersection between the ground truth and the machine segmentation regions over their union. It ranges from 0% to 100% of similarity. VOE is the JSC correponding error measure. Finally, to measure the segmentation accuracy in terms of distance betwen the predicted segmentation boundary and the ground truth, Hausdorff distances using Euclidean distance are used.

## 3. Results and Discussion

### 3.1. Performance Analysis

The 58 volumetric CT and MR images were randomly divided into 49 for training, 9 for validation/testing. Table 2 presents the statistical analysis of the 9 test images after being trained and tested 10 times for 600 epochs. DSCs, SVDs, JSCs, VOEs, and HDs, are calculated from the gold standard labels and predicted labels. DSCs of the skull varies from 0.6847 to 0.8056, with a mean ± SD of 0.7300 ± 0.04, and from 0.9654 to 0.9833 for background, with a mean ± SD of 0.9740 ± 0.007.

To improve the results, by a reduction of regions of non-interest, a 3D UNet task was performed by using the same software/equipment previously used. The difference between these two methods, rely on the creation of the gold standard from different image modalities and 3D UNet parameters. This approach does not require the CT and MRI scans to overlap in order to create MRI gold standard labels. Instead, this approach uses it own volumetric MRI to create the labels through the same processing presented previously.

Using the same datasets from the cancer imaging archive data collection, 62 different volumetric MRI were used to create the brain dataset, where 53 were used for training, 3 for validation, and 6 for testing purpose. Table 3 shows the CNN implementation details of the brain segmentation and the statistical analysis of the 6 tested images are presented in the Table 4 after 10 rounds of testing. DSCs, SVDs, JSCs, VOEs, and HDs, are calculated from the brain gold standard labels and brain predicted labels.

These results show an accuracy rate of 0.9244 ± 0.04, however, the brain must be perfeclty extracted. Therefore, after CNN was tested on the 58 initial volumetric MRI, the labels generated in this process were manually corrected using the Matlab program created by [49] in order to optimize brain removal.

After removing the gray and white matter, the modified 58 volumetric images, 49 for training and 9 for validation/testing, were then presented to the CNN using the same parameters shown in Table 1, and the statistical analysis of the 9 tested images are shown in Table 5 and Table 6.

From Table 5 and Table 6, it can be stated that the reduction in the information contained in the images, such as the removal of the brain, helps in improving the segmentation of the skull bones. In fact, the DSC improvement varied from 2.31% to 7.27% for the skull and 0.24% to 0.99% for the background, which demonstrates that the removal of information in images inherently affects the segmentation of the skull directly. Thus, the initial DSC mean for skull and background improved from 0.7300 ± 0.04 and 0.9740 ± 0.007 to 0.7826 ± 0.03 and 0.9804 ± 0.004 respectively, with a decrease in the standard deviation.

The results represented by DSCs from Table 2 in this present study are marginally lower to those reported in [15,16,17,18], who achieved mean DSCs of (0.7346, 0.6918, 0.6337), 0.75, 0.75, and (0.7344, 0.7446) respectively, and lower than that reported by [21] of (0.76 and 0.80). Howerver, the interpretations of the different results found in these studies must be evaluated with caution due to the differences between the databases used, computational methods, and so forth.

This distinction may be attributed to the size of the dataset used. [40,50] reported a mean dice coefficient of 0.9189 and 0.9800 for CT skull segmentation using CNN when a dataset of 195 and 199 images was used, and [50] attributed this high DSC to the dataset size and image resolution when compared to other study ([25]). Thus, a change in the size of the dataset may contributed to the improvement of the values of the DSCs. In addition, the geometric disparity, variations, and deformity between the skulls sets become more evident as the dataset increases. As the related works used small datasets, this aspect may have led to the high DSCs reported.

Furthermore, we used four distinct datasets [31,32,35,36] that use different CT and MRI devices with a variety of parameters. These datasets included variations in age, ethnicity, and medical history. In addition, several patients have undergone cephalic surgical treatment which may altered the skeletal structure of the skull. In total, 40 images have part of the skull removed due to brain abnormalities. These removals may have affected the performance of the segmentaion.

From Table 6, JSC and HD improved from the initial values (Dataset 1), while SVD and VOE decreased. These improvements and reductions can be due to the fact that there is less overlap between the ground truth and the predicted segmentation in the brain region since there is no brain.

One drawback of presented method is during the creation of the gold standard STL model. An expert manually corrected the models by edge smoothing or noise residue removal which may prompt the CNN to learn the defects the expert may have created. Furthermore, during the conversion from STL model into label (voxelisation), a quantity of information from the skull-voxel may have been erroneously labeled as background when converted into imaging-voxel. Other disadvantage regards the number of training images. Unfortunately, the amount of usable data that can be acquired from the same patient for both CT and MR images is difficult because of alignment issues, and commonly limited due to ethical and privacy considerations and regulations.

The results found in this article reflect a long-standing search for the development of a technique for bone segmentation in MRI, however, the proposed method DSC (0.7826 ± 0.03) does not exceed the performance of current CT techniques, DSC of 0.9189 ± 0.0162 [40]), DSC of 0.9200 ± 0.0400 [25]), and DSC of 0.9800 ± 0.013 [50]).

### 3.2. Comparison between UNet, UNet++, and UNet3+

Further comparison is necessary to see how the DSC behave in various deep learning methods. UNet was compared to UNet++ [51], an encoder-decoder network where a series of dense skip pathways are connected in the encoder and decoder sub-networks, and UNet3+, a deep learinng approach that uses the full-scale aggregated feature map to learn hierarchical representations while, using feature maps in various scales, incorporate low-level details with high-level semantics [52]. Table 7 compares UNet, UNet++, and UNet3+ architecture in terms of segmentation accuracy measured by dice similarity coefficient on both datasets 1 and 2. The parameters for each CNN were identical, with an encoder depth of 3, a mini batch size of 16, an initial learning rate of 0.005, and the training was carried out in 100 periods.

As seen, UNet3+ outperformed UNet and UNet++, obtaining average improvement over UNet of 1.51% and 0.26% in datasets 1 and 2. The UNet algorithm took about an hour to train the 100 epochs, and UNet3+ took about 2.5 times longer. Therefore, if the training time of the UNet3+ is disregarded, this network may be used to slightly improve segmentation results.

## 4. Conclusions

This study presents a 3D CNN developed for skull segmentation in MRI where the trained labels were acquired from the same patient CT scans in standard tessellation language. This method initially demonstrated a skull DSC overlap of 0.7300 ± 0.04 and 0.9740 ± 0.007 for background, however, after the removal of the gray and white matters, DSC reached an average of 0.7826 ± 0.03 and 0.9804 ± 0.004 respectively. Due to the limited number of datasets tested, further research may be undertaken to improve the mean DSC. In summary, the present method is a step forward in the improvement of bone extraction in MRI using CNN to achieve average DSC rates similar to those obtained in CT scans.

## Figures and Tables

**Table 1:** Skull Segmentation Implementation Details.

Parameter	Value
Optimizer	Adam
Encoder Depth	4
Filter Size	3
Number of First Encoder Filters	15
Patch Per Image	1
Mini Batch Size	128
Initial Learning Rate	5 × $10^{-2}$

**Table 2:** Statistical Analysis of the first dataset.

DSC (Skull)	DSC (Background)	SVD (Skull)	JSC (Skull)	JSC (Background)	VOE (Skull)	HD (Skull)
0.8056±0.02	0.9833±0.001	0.1944±0.02	0.6746±0.02	0.9672±0.003	0.3254±0.02	18.25±0.81
0.7706±0.02	0.9805±0.002	0.2294±0.02	0.6267±0.02	0.9618±0.003	0.3733±0.02	23.48±3.44
0.7667±0.02	0.9807±0.002	0.2333±0.02	0.6217±0.02	0.9622±0.003	0.3783±0.02	15.41±3.18
0.7366±0.01	0.9779±0.001	0.2634±0.01	0.5830±0.01	0.9568±0.001	0.4170±0.01	19.66±2.32
0.7177±0.01	0.9731±0.001	0.2823±0.01	0.5597±0.01	0.9476±0.001	0.4403±0.01	40.01±3.33
0.7014±0.02	0.9673±0.001	0.2986±0.02	0.5401±0.03	0.9366±0.001	0.4599±0.03	19.38±3.29
0.6940±0.01	0.9672±0.001	0.3060±0.01	0.5314±0.01	0.9366±0.001	0.4686±0.01	28.41±0.57
0.6917±0.02	0.9654±0.001	0.3083±0.02	0.5287±0.02	0.9331±0.002	0.4713±0.02	39.65±3.00
0.6847±0.01	0.9707±0.001	0.3153±0.01	0.5206±0.01	0.9430±0.001	0.4794±0.01	34.85±2.93
0.7300±0.04	0.9740±0.007	0.2700±0.040	0.5763±0.051	0.9494±0.012	0.4237±0.051	26.57±8.98

**Table 3:** Brain Segmentation Implementation Details.

Parameter	Value
Optimizer	Adam
Encoder Depth	3
Filter Size	5
Number of First Encoder Filters	7
Patch Per Image	2
Mini Batch Size	128
Initial Learning Rate	$10^{-3}$

**Table 4:** Statistical Analysis of the brain segmentation.

DSC (Brain)	DSC (Background)	SVD (Brain)	JSC (Brain)	JSC (Background)	VOE (Brain)	HD (Brain)
0.8547±0.02	0.9724±0.001	0.1453±0.02	0.7465±0.03	0.9463±0.001	0.2535±0.03	22.59±4.63
0.9053±0.01	0.9769±0.001	0.0947±0.01	0.8270±0.01	0.9548±0.003	0.1730±0.01	13.96±0.40
0.9436±0.01	0.9910±0.002	0.0564±0.01	0.8934±0.02	0.9821±0.003	0.1066±0.02	10.91±1.84
0.9464±0.01	0.9894±0.002	0.0536±0.01	0.8984±0.02	0.9790±0.003	0.1016±0.02	11.41±0.63
0.9469±0.01	0.9887±0.002	0.0531±0.01	0.8992±0.02	0.9776±0.005	0.1008±0.02	17.78±1.95
0.9491±0.02	0.9888±0.004	0.0509±0.02	0.9035±0.04	0.9778±0.008	0.0965±0.04	14.89±2.34
0.9244±0.04	0.9845±0.008	0.0756±0.04	0.8613±0.06	0.9696±0.015	0.1387±0.06	15.26±4.37

**Table 5:** Statistical Analysis of the second dataset.

DSC (Skull)	DSC (Background)	SVD (Skull)	JSC (Skull)	JSC (Background)	VOE (Skull)	HD (Skull)
0.8288±0.03	0.9857±0.002	0.1712±0.03	0.7076±0.01	0.9719±0.003	0.2924±0.01	09.89±1.58
0.8095±0.01	0.9845±0.001	0.1905±0.01	0.6800±0.01	0.9695±0.001	0.3200±0.01	11.05±0.50
0.8038±0.01	0.9846±0.001	0.1962±0.01	0.6719±0.01	0.9696±0.001	0.3281±0.01	12.32±0.43
0.8052±0.03	0.9839±0.003	0.1948±0.03	0.9684±0.005	0.6739±0.04	0.3261±0.04	10.69±2.25
0.7904±0.01	0.9812±0.001	0.2096±0.01	0.9631±0.003	0.6534±0.01	0.3466±0.01	18.33±0.92
0.7587±0.01	0.9758±0.001	0.2413±0.01	0.9528±0.001	0.6112±0.09	0.3888±0.09	14.09±0.31
0.7627±0.01	0.9756±0.001	0.2373±0.01	0.9523±0.003	0.6164±0.01	0.3836±0.01	14.50±1.33
0.7532±0.03	0.9753±0.003	0.2468±0.03	0.9518±0.006	0.6042±0.03	0.3958±0.03	23.71±1.71
0.7310±0.02	0.9772±0.001	0.2690±0.02	0.9554±0.001	0.5760±0.03	0.4240±0.03	24.91±1.74
0.7826±0.03	0.9804±0.004	0.2174±0.03	0.6439±0.041	0.9617±0.01	0.3561±0.04	15.50±5.28

**Table 6:** Differences between Dataset 2 minus Dataset 1.

DSC (Skull)	DSC (Background)	SVD (Skull)	JSC (Skull)	JSC (Background)	VOE (Skull)	HD (Skull)
0.0231	0.0024	−0.0231	0.0046	0.0331	−0.0331	−08.36
0.0390	0.0040	−0.0390	0.0077	0.0533	−0.0533	−12.43
0.0371	0.0038	−0.0371	0.0074	0.0503	−0.0503	−03.09
0.0686	0.0060	−0.0686	0.0116	0.0909	−0.0909	−08.96
0.0727	0.0081	−0.0727	0.0155	0.0937	−0.0937	−21.68
0.0573	0.0086	−0.0573	0.0162	0.0711	−0.0711	−05.29
0.0687	0.0083	−0.0687	0.0157	0.0850	−0.0850	−13.91
0.0616	0.0099	−0.0616	0.0187	0.0755	−0.0755	−15.94
0.0463	0.0065	−0.0463	0.0124	0.0555	−0.0555	−09.94
0.0527	0.0064	−0.0527	0.0122	0.0676	−0.0676	−11.07

**Table 7:** Comparison between UNet, UNet++, and UNet3+ in four samples.

	Dataset 1			Dataset 2		
Samples	Unet	Unet++	Unet3+	Unet	Unet++	Unet3+
**A**	0.6913±0.003	0.5670±0.011	0.7235±0.050	0.8144±0.005	0.5915±0.015	0.8141±0.003
**B**	0.6545±0.013	0.6292±0.016	0.6589±0.005	0.7462±0.004	0.6393±0.008	0.7567±0.003
**C**	0.7068±0.005	0.5835±0.087	0.7194±0.004	0.8562±0.007	0.6672±0.017	0.8560±0.006
**D**	0.6886±0.005	0.5189±0.091	0.6997±0.002	0.7500±0.003	0.6315±0.008	0.7503±0.005

## Data Availability

The datasets presented in this study, are openly available at: TCIA—https://www.cancerimagingarchive.net/ (accessed on 1 March 2021).

## Abbreviations

The following abbreviations are used in this manuscript:

CNN	convolutional neural network
CR	Computed Radiography
cRBM	convolutional restricted Boltzmann machines
CSF	cerebro-spinal fluid
CT	computed tomography
DSC	dice similarity coefficient
FN	false negatives
FP	false positives
GB	gigabyte
HD	hausdorf distances
JSC	jaccard similarity coefficient
MRI	magnetic resonance imaging
PT or PET	positron emission tomography
ROI	regions of interest
RT	radiotherapy
RTDOSE	radiotherapy dose
RTPLAN	radiotherapy plan
RTSTRUCT	radiotherapy structure set
SC	secondary capture
SD	standard deviation
SPM8	statistical parametric mapping 8
STL	standard tessellation language
SVD	symmetric volume difference
SVM	support vector machine
TP	true positives
VOE	volumetric overlap error
VRAM	video random access memory
